# Rapid Detection of Amyloid β1–42 via PAMAM G4 Supported Molecularly Imprinted Sensor

**DOI:** 10.1021/acsomega.6c01809

**Published:** 2026-04-18

**Authors:** Hilmiye Deniz Ertuğrul Uygun, Münire Nalan Demir

**Affiliations:** † 37508 Dokuz Eylül University, Center for Fabrication and Application of Electronic Materials, Izmir 35390, Turkey; ‡ 37508 Dokuz Eylül University, Faculty of Science, Department of Chemistry, Izmir 35390, Turkey

## Abstract

In this research, a novel sensor system was prepared for the rapid and selective determination of Amyloid β1–42 (Aβ1–42), a key biomarker in the early recognition of Alzheimer’s disease. A screen-printed gold electrode (AuE) was used as the base for a self-assembled monolayer (SAM)-based molecularly imprinted polymer (MIP). The electrode was modified by sequential incubation in 100 mM cysteamine, 5% glutaraldehyde, and PAMAM G4 dendrimer. Electropolymerization was performed using pyrrole-3-carboxylic acid and Aβ1–42. Desorption was achieved using 500 mM HCl to remove the template molecule, generating specific recognition sites. The sensor’s performance was determined by electrochemical impedance spectroscopy (EIS) and cyclic voltammetry (CV), with additional surface characterization via SEM, XPS, and FT-IR. The nonimprinted polymer (NIP) showed no response to Aβ1–42, confirming the selectivity of the MIP. Selectivity tests using Tau protein showed minimal interference. The quantification limit (LOQ) and the limit of detection (LOD) were found to be 0.42 and 0.14 ng/mL, respectively. In diagnostic applications, the proposed sensor shows promise as a quick, sensitive, and selective way to detect Aβ1–42.

## Graphical abstract

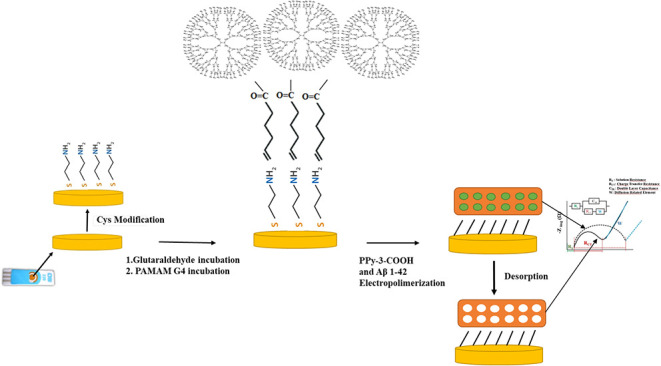

## 1 Introduction

Alzheimer’s disease (AD) is the most common form of dementia and one of the leading causes of death worldwide. It is a steady, irreversible neurological disease that affects everyday functioning, memory, and mental abilities. As the disease advances, neuronal damage intensifies, leading to a significant decline in quality of life. Despite extensive research, there is currently no cure for AD, and developing treatments only provide limited symptomatic relief.^1^ After heart disease, stroke, and cancer, this condition is the biggest cause of death for people over 65 in developed nation.^2^ Currently, the diagnosis of AD is achievable through the assessment of biomarkers like Amyloid β42 (Aβ-42) Tau, Phosphorylated Tau, Dopamine, and α-Synuclein, which exhibit variations in physiological fluids throughout the progression of the illness.^3^ People’s life expectancy and standard of living have naturally grown as a result of medical and technological advancements that have made it easier to diagnose and cure a variety of diseases. Therefore, the number of elderly people has increased. The challenges associated with aging have likewise increased with the number of senior people.^4^

The histological hallmark of AD is the development of Aβ plaque. The process by which Amyloid Precursor Protein (APP) splits into two isoforms produces Aβ, including Aβ40 and Aβ-42. Aβ-42 is more cytotoxic than Aβ-40 and has been identified as a major component of amyloid plaques. AD etiology is intimately linked to Aβ-42 agglomeration and plaque build up in the brain tissue.^5,6^ For the determination of Aβ-42 biomarkers, The Aβ-42 biomarkers are determined by ELISA (enzyme-linked immunosorbent method),^7^ MS (mass spectrometry),^8^ and SPR (surface plasmon resonance).^9^ Due to the time-consuming nature of current procedures and the high cost of consumables and equipment, the hunt for innovative approaches is still ongoing. A sensor device that can swiftly, selectively, precisely, and affordably detect this chemical has been developed in our work.

Because biosensors are quick, accurate, affordable, and useful enough for patients to use themselves, they hold promise in this area. To develop biosensors, which are tiny analyzers, a biorecognition receptor—such as an enzyme, antibody, receptor, protein, or DNA molecule that only shows affinity for the analyte molecule—can be mounted on a physicochemical transducer.^10^ Medical applications for biosensor systems include environmental pollution monitoring, food safety and content determination, and cancer detection. Because biological receptors are particular for the analytes, biosensor systems seem to be very beneficial and sensitive measuring methods. However, the employment of biological receptors as detecting molecules restricts the analysis in various environmental situations.^11,12^ Biological molecules require optimal operating conditions in order to perform efficiently. This includes the pH, polarity, temperature, and ion strength of the measuring liquid. To get around these restrictions, synthetic affinity-based detection systems can be created.^13,14^ These techniques make it possible to create sensing materials that are more stable and less susceptible to environmental influences. In order to create these synthetic receptors, monomers with unique functional groups are polymerized such that they encircle the analyte molecule in accordance with its three-dimensional characteristics. Therefore, specific molecules can be able to enter specialized cavities that can be designed on these polymers. Molecular imprinting, often known as molecular imprinting technology (MIT), is a method for creating artificial receptors. These created polymers are also known as molecularly imprinted polymers, or MIPs.^15^ By using these polymers in biosensor systems, the nature of biosensors that are susceptible to external factors can be eliminated.^16^ In biomarker diagnostics, electroanalytical techniques have garnered a lot of attention because of their intrinsic simplicity, high sensitivity, and speed. In the 1960s, electrochemical sensors were initially created.^17^ The measurement principle of electrochemical biosensors is to measure the electrical signals produced on electrode surfaces to determine the binding of biomolecules.^18^ Because of their exceptional qualities and wide range of applications, electrochemical sensors are unique in the field of analytical sensing. Their remarkable sensitivity and selectivity are among their key benefits.^19^ The development of quick, sensitive, and affordable sensors for Aβ1–42 detection is crucial because of its crucial involvement in AD pathogenesis. Here, we introduce a new electrochemical biosensor that uses a generation 4 (G4) polyamidoamine (PAMAM) dendrimer to enhance a molecularly imprinted polymer (MIP). PAMAM dendrimers were selected as functional nanoscaffolds owing to their highly branched three-dimensional structure and high density of terminal amine groups. These features significantly increase the effective surface area and provide abundant active sites, thereby enhancing molecular imprinting efficiency, facilitating template–monomer interactions, and ultimately improving the sensitivity of the sensing platform. This approach leverages the specificity of molecular imprinting and the signal amplification capabilities of dendrimers to create a highly selective and sensitive platform for Aβ1–42 measurement.

## 2 Materials and Methods

### 2.1 Chemicals and Consumables

All chemicals and biologicals were acquired from Sigma-Aldrich (SA). A three-electrode setup (carbon working electrode, platinum counter electrode, and Ag/AgCl reference electrode), the DRP250 gold screen-printed electrodes (SPE) were acquired from the Dropsense company (Spain).

### 2.2 Instruments and Measurements

The electrochemical measurements were performed using a potentiostat of the Palme Sense 3 series. The measurements were performed and the impedance curve was calculated using the PSTrace 5.7 interface application. A screen-printed electrode system that integrates the working, reference, and counter electrode triple systems into a single electrode was used for electrochemical measurements (EIS, CV). EIS curves were calculated by using the circuit model as shown Figure S1A Thermo Scientific K-Alpha model X-ray photoelectron spectroscopy (XPS) and a COXEM EM30 brand scanning electron microscope (SEM) were used to evaluate the electrode surface. For the XPS measurement, a monochromatic Al–Kα (1486.7 eV) X-ray source was employed. With a scanning speed of 1 eV and a pass energy of 150 eV, the XPS survey scan was performed between −10 and 1350 eV. FT-IR spectra of the electrodes were monitored by a spectrometer (Thermo Scientific Nicolet iS10) in the spectral range 4000–450 cm^–1^.

### 2.3 Preparation of Amyloid β Sensor

The gold electrode’s surface was modified to create the recognition surface for the measurement of β amyloid (Aβ 1–42). The electrode was submerged in 100 mM cysteamine (Cys) for 2 h in order to accomplish surface modification. After this period, the electrode was thoroughly cleaned with pure water and incubated for 45 min in an environment containing 5% glutaraldehyde. The electrode was then immersed in PAMAM G4 dendrimer solution for an hour. After that, pyrrole 3 carboxylic acid (PPy-3-COOH) and Aβ 1–42 (template molecule) were used for electropolymerization on the electrode. CV and EIS measurements were performed in Fe (CN)_6_^3–/4–^ following each polymerization step, excluding the glutaraldehyde incubating stage. These procedures were performed to characterize the immobilization steps. CV experiment conditions were chosen as follows: −0.2 to 0.5 V at a 100 mV/s scan rate. The EIS measurement was performed using 180 mV DC and 10 mV AC between 10,000 and 0.05 Hz. The electropolymerization was carried out by cyclic voltammetry in the potential range of 0 to 1.2 V at a scan rate of 100 mV s^–1^ for 10 cycles. The template was pulled out after the polymerization process. As a result, among the 100 and 500 mM HCl solutions, 500 mM HCl was chosen as the desorption agent for 30 min. Aβ 1–42 standard solutions were used to generate standard graphics once the template was removed. To do this, a number of standard solutions were made, 20 μL of each was put onto the electrode surface, and after 30 min of incubation, CV and EIS measurements were made. Reproducibility, repeatability, selectivity, limits of determination (LOD and LOQ), and real sample testing were performed in the performance steps in accordance with the calibration graph’s linear measurement range.

## 3 Results and Discussion

The gold electrode was modified to create the recognition surface for the measurement of β amyloid (Aβ 1–42). The electrode was maintained in 100 mM cysteamine solution (Cys) for this purpose. After that, pyrrole 3-COOH and Aβ 1–42 were used to electropolymerize the electrode while it was treated with a PAMAM G4 dendrimer (Figure 1). CV and EIS data were collected following each polymerization phase, and the presence or absence of polymerization was assessed (Figure 2A,B). A calibration graph was created using solutions in the range of 0.5–20 ng/mL once the polymerization was determined to be successful (Figure 2). To characterize the electrode surface, SEM, FT-IR and XPS analyses were performed.

**Figure 1:**
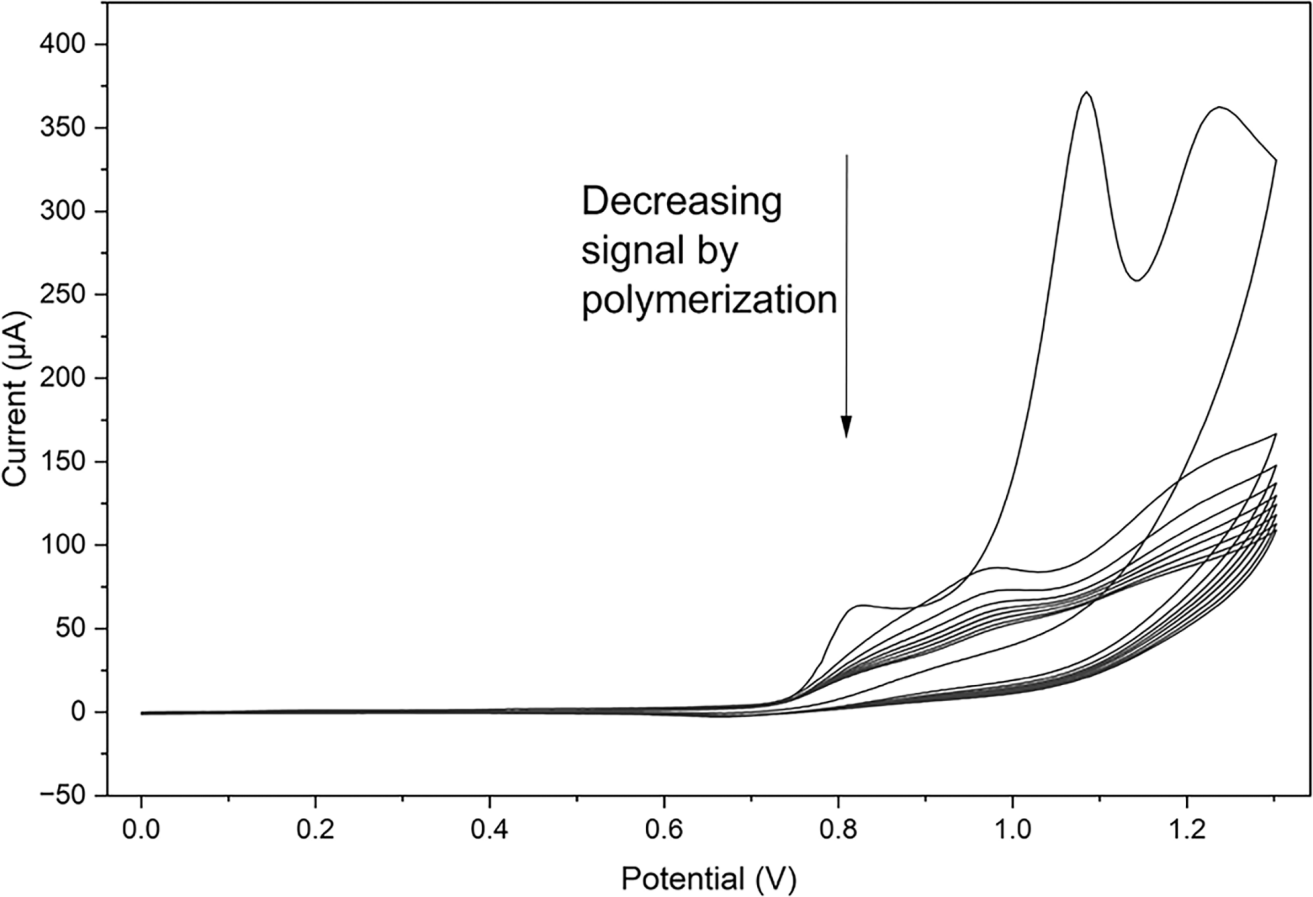
Performing electropolymerization of the monomer around amyloid-β by cyclic voltammetry.

**Figure 2:**
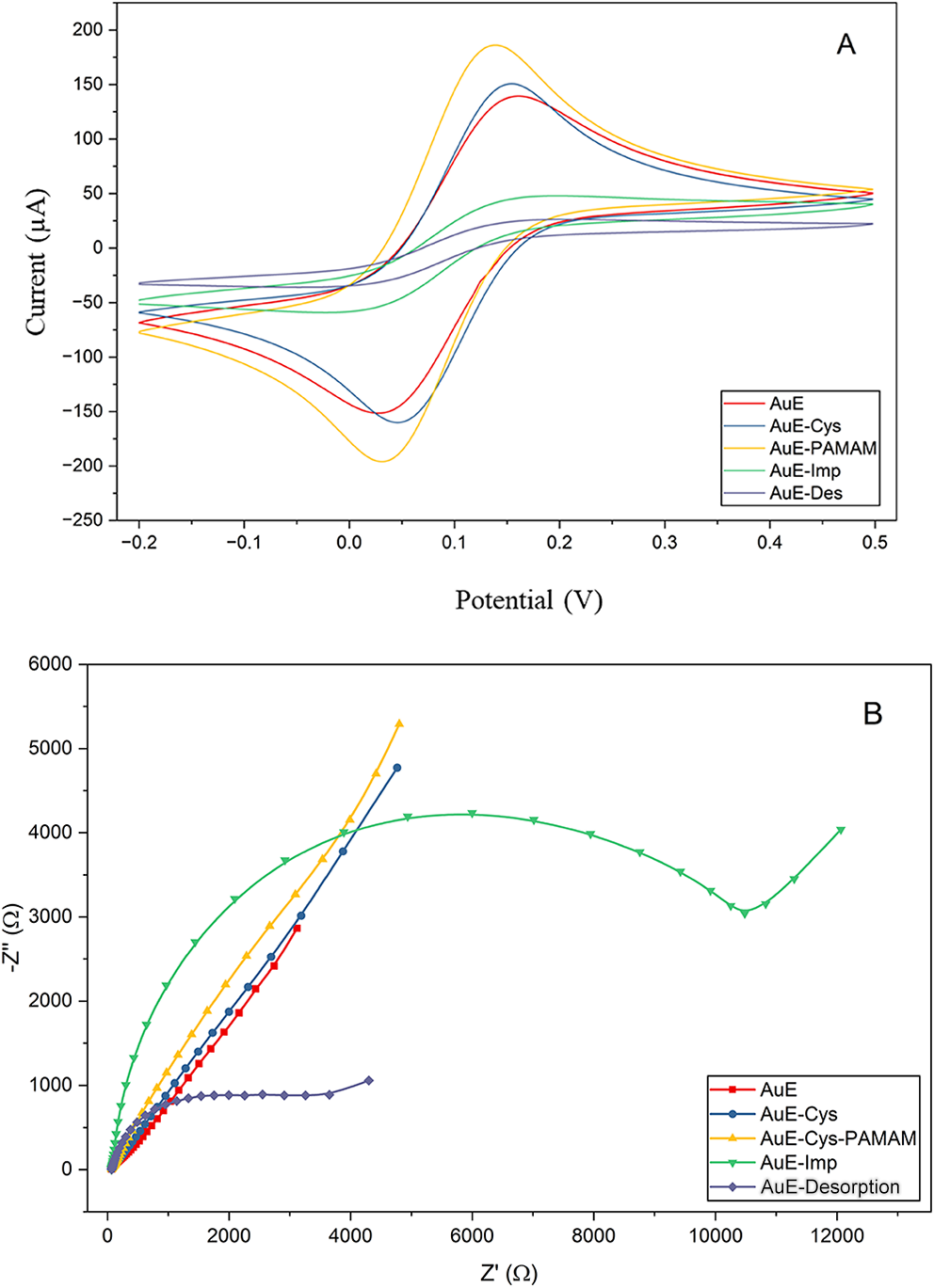
(A) Cyclic voltammetry (CV) responses demonstrating sequential electrode modification steps. Red: bare AuE; Blue: AuE–Cys; Yellow: AuE–Cys–PAMAM G4; Purple: AuE–Cys–PAMAM–Aβ_1–42_–PPy_3_COOH, and Green: desportion (B) Electrochemical impedance spectroscopy (EIS) Nyquist plots illustrating sequential electrode modification steps: Red: bare AuE; Blue: AuE–Cys; Yellow: AuE–Cys–PAMAM G4; Green: AuE–Cys–PAMAM–Aβ_1–42_–PPy_3_COOH; Purple: electrode response after the desorption step.

### 3.1 Characterization of the Electrode

Using EIS, CV, SEM, FT-IR, and XPS equipment, surface characterization analyses of the constructed sensor system were carried out. To keep an eye on the electrodes’ surface morphology, SEM examination was done. A small layer of gold was applied to the electrodes for SEM examination in order to improve analysis. An ion coater device was employed for this. SEM examination was carried out with a 20 kV application voltage and high vacuum. Twelve was found to be the ideal spot size for the required magnifications. XPS analysis was used in the study to ascertain chemical properties of the surface and elemental composition. The XPS measurement was performed using a monochromatic Al–Kα (1486.7 eV) X-ray source with a 400 nm diameter beam. With a scanning speed of 1 eV and a pass energy of 150 eV, the range of the XPS survey scan was −10 to 1350 eV.

At each stage of electrode modification, the interfacial characteristics and electron transfer resistance (*R*_ct_) were examined using EIS. Figure 2 displays the Nyquist plots, which show the charge-transfer kinetics between the redox probe and the electrode surface. In the high-frequency region, the semicircle diameter is equal to *R*_ct_. Because of its clean and conductive surface, the bare gold electrode (AuE, red curve) showed a small semicircle, suggesting a low *R*_ct_ and easy electron transmission. When cysteamine (AuE–Cys, blue curve) was added, the semicircle’s diameter expanded compared to bare Au electrode. The development of a compact cysteamine monolayer via Au–S bonding, which creates a partly insulating organic barrier that prevents charge transfer at the interface, is responsible for this rise. *R*_ct_ increased more after the PAMAM G4 dendrimer (AuE–Cys–PAMAM G4, yellow curve) was attached. The thick and resistive coating formed by PAMAM’s highly branched and amine-rich dendritic structure increases steric hindrance and restricts the migration of redox species to the electrode surface. Following the immobilization of Aβ1–42 and conductive PPy3COOH (AuE–Cys–PAMAM–Aβ1–42–PPy3COOH, yellow curve), a notable increase in the semicircle diameter was observed compared to the previous step. The thick/insulating polymer and protein layer formed on the surface severely hinders the direct electron transfer of the [Fe (CN)_6_] ^3–/4–^ redox couple to the electrode, thus significantly increasing the charge transfer resistance (*R*_ct_). This finding supports the success of immobilization and the decrease in the steric/electrochemical accessibility of the surface. The semicircle diameter further shrank following template removal (green curve), indicating that Aβ1–42 was successfully desorbing and that molecularly imprinted cavities had formed on the electrode surface. By enhancing electron diffusion and electrolyte access, the creation of these imprinted spots lowers the total impedance. The successful construction of the MIP-based sensing interface and the creation of particular binding sites for Aβ1–42 recognition are strongly supported electrochemically by the progressive changes in *R*_ct_ values throughout each modification stage.

Cyclic voltammetry (CV) measurements were performed to track the gold electrode’s surface modification step-by-step and assess how the electron transfer properties changed at each production stage. With a low peak-to-peak potential separation (Δ*E*_p_) and the largest anodic and cathodic peak currents, as seen in Figure 3, the bare gold electrode (AuE, pink curve) demonstrated a clean surface with quick electron transfer kinetics. There was a noticeable drop in the redox current with the addition of cysteamine (AuE–Cys, green curve). This decrease is explained by the Au–S bonding-induced self-assembled monolayer that forms on the Au surface, where the carboxyl and terminal amino groups of cysteamine function as a partially insulating barrier to prevent charge transfer. The successful immobilization of the dendritic macromolecule was confirmed by an additional decrease in current response upon further functionalization with the PAMAM G4 dendrimer (AuE–Cys–PAMAM G4, red curve). PAMAM’s dense amine-terminated structure creates a larger organic layer at the electrode interface by increasing the steric hindrance and electron-transfer resistance. The interaction between the conductive properties of polypyrrole and the insulating peptide layer caused a noticeable change in the CV signal after the addition of Aβ1–42 and the conductive polymer PPy_3_COOH (AuE–Cys–PAMAM–Aβ1–42-PPy_3_COOH, blue curve). The coexistence of both biomolecular and electroactive components on the electrode surface was reflected in the altered current response that was the result of the combined effect. Redox peak currents significantly increased during the desorption procedure (orange curve), which involved removing the template Aβ1–42 molecules from the polymer matrix. The successful creation of molecularly imprinted cavities, which promote the diffusion of redox species and partially restore electron transfer, is demonstrated by this current recovery.

**Figure 3:**
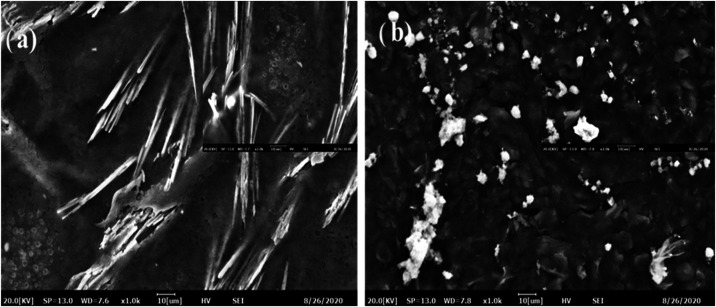
SEM images of Aβ 1–42 sensor system. (a) MIP sensor ×1000 and ×2000 magnification (b) NIP sensor ×1000 and ×2000 magnification.

The results obtained using the CV method were used to calculate the electrochemically active surface area. The necessary calculations were performed using the Randles–Sevcik equation^20^ and showed in Table S1. The electrochemical surface area (ECSA) values were estimated from cyclic voltammetry measurements in 50 mM Fe (CN)_6_^3–/4–^ solution at a scan rate of 100 mV s^–1^ using the Randles–Sevcik equation. The increase in ECSA upon PAMAM alteration validates its function as a nanoscaffold offering a high density of active sites, whereas the subsequent decline during MIP formation is ascribed to the polymeric matrix partially blocking the electroactive surface.

In summary, the successful manufacturing of the MIP-based sensing interface and the successful imprinting of Aβ1–42 within the polymeric network are demonstrated electrochemically by the successive changes in current magnitude and Δ*E*_p_ throughout the modification processes.

Figure 3 shows SEM pictures of the MIP and NIP (nonimprinted) sensor surfaces. In the MIP sensor, It is evident in MIP that the protein enters the polymeric structure and modifies the polymer. The surface morphologies of the molecularly imprinted polymer (MIP) and nonimprinted polymer (NIP) electrodes were investigated via scanning electron microscopy (SEM) at a magnification of ×1000. As shown in Figure 4, the MIP-modified electrode surface (Figure 3a) exhibits prominent needle-like, elongated structures distributed across the surface. These characteristics imply that a porous and well-organized polymer network was successfully formed, most likely as a result of amyloid β (Aβ) acting as a template molecule during the polymerization process. The formation of selective recognition cavities complementary to the size and shape of Aβ is indicated by the observed morphology. On the other hand, the NIP electrode surface (Figure 3b), which was made with the same setup but without the Aβ template, exhibits a notably different shape. There are no distinct porous or directed structures, and the surface seems smoother and more compact with globular aggregates that are amorphous and dispersed randomly. This suggests that the polymerization occurs nonspecifically in the absence of the template’s guiding impact, producing a more uniform and less useful surface.

**Figure 4:**
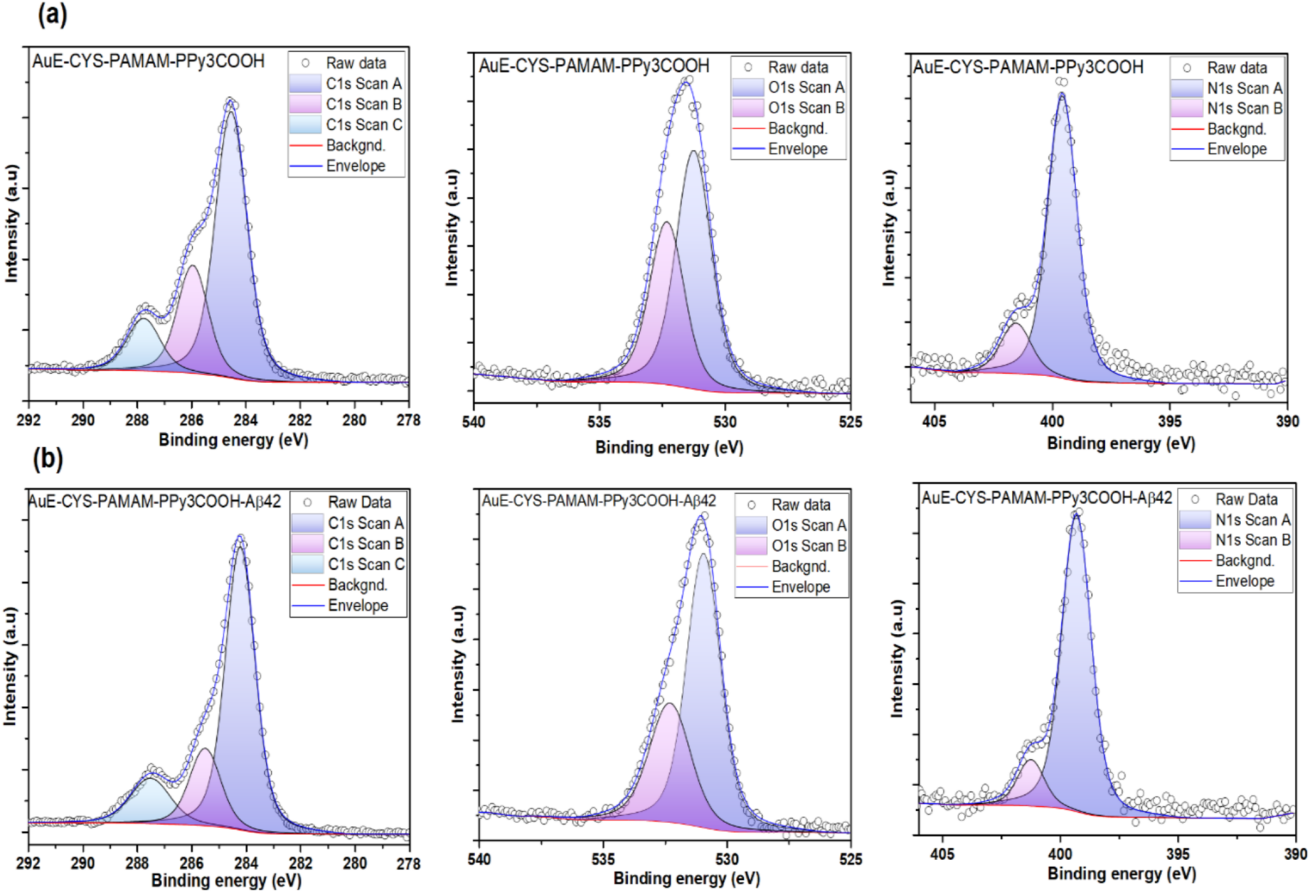
XPS deconvolution peaks of (a) Au-CYS-PAMAM and (b) Au-CYS-PAMAM-Aβ42.

The effective imprinting of Aβ in the MIP structure is confirmed by these morphological variations. While the NIP acts as a crucial control for evaluating nonspecific interactions, the MIP’s template-induced cavities and rougher surface are anticipated to improve the target analyte’s selective rebinding.

After every electrode preparation step, an XPS analysis was performed (Figure S2). The ratio of elements distribution for each electrode is summarized in Table 1. The results of this evaluation showed that the electrode preparation and immobilization procedures were executed effectively. It was observed that the gold ratio in the bare gold electrode decreased after each step for electrode surface immobilization. However, the amounts of the elements C, N, and O changed with each molecular structure that was added to the surface.

**Table 1:** % Weight of Elements for Each Stage of the Electrodes Preparation

**electrode**	**% weight Au**	**% weight C**	**% weight O**	**% weight N**
AuE	57.70	12.16	7.64	-
AuE-CYS	48.64	33.61	12.32	5.43
AuE-CYS-PAMAM	4.32	59.91	21.84	13.93
AuE-CYS-PAMAM-Aβ42	-	66.15	22.64	11.22
AuE-CYS–PAMAM-PPy3COOH	2.26	53.69	19.43	11.85

High-resolution XPS analyses were performed to elucidate the chemical changes on the electrode surface after imprinting with Aβ1–42. Figure 4a,b is represented of XPS deconvolution peaks of the NIP electrode (Au-CYS-PAMAM) and the MIP electrode (Au-CYS-PAMAM-Aβ42). The C 1s spectra of the AuE–CYS–PAMAM–PPy_3_COOH electrode (without Aβ1–42) exhibited three major components corresponding to C–C/C–H at ∼284.8 eV, C–N/C–O at ∼286.0 eV, and a minor carbonyl peak at ∼288.2 eV. After incorporation of Aβ1–42 (AuE–CYS–PAMAM–PPy3COOH–Aβ1–42), the relative intensity of the C=O component markedly increased, together with a more pronounced C–N/C–O contribution, consistent with the peptide backbone’s carbonyl and amide groups.

Similarly, the O 1s region in the Aβ-free electrode was characterized by a dominant peak at ∼531.5 eV (carbonyl oxygen) and a smaller contribution at ∼533 eV (C–O/adsorbed water). Upon Aβ1–42 loading, the carbonyl-related component became more prominent, confirming the presence of additional amide oxygen functionalities from the peptide. The N 1s spectra further supported these findings: while the Aβ-free electrode displayed signals at ∼399.5 eV (amine/–NH2) and ∼401.0 eV (protonated amine/amide), the Aβ-loaded electrode exhibited an enhanced amide contribution around 400–401 eV. This increase indicates the incorporation of peptide amide nitrogen and demonstrates strong interactions between Aβ1–42 and the PAMAM/polypyrrole matrix, most likely through hydrogen bonding and electrostatic interactions. Overall, the observed increases in C=O, C–N, and amide N components after Aβ1–42 incorporation, combined with consistent O 1s features, provide compelling evidence of successful peptide binding to the imprinted polymer layer. These XPS results are in excellent agreement with FT-IR findings, confirming both the effective imprinting of Aβ1–42 and the chemical stability of the underlying sensor platform.

The FTIR spectra of MIP electrode and the electrode after desorption are shown in Figure 5. The FT-IR spectra of the prepared electrodes provided clear evidence for the successful imprinting and subsequent removal of Aβ1–42. In the FT-IR spectrum, the large OH band observed around 3300 cm^–1^ indicates the increase of OH groups by both Aβ42 and the polymer matrix. In the presence of Aβ1–42 this band is broad and strong, due to the rich hydrogen bonding from the peptide. After desorption of the Aβ1–42, this peak becomes weaker, indicating a drop in hydrogen bonding density once Aβ42 is removed. The saturated C–H stretching band at ∼2940 cm^–1^ refers to aliphatic chain vibrations from pyrrole-3-carboxylic acid and C=O/C–O stretching at ∼1700 and 1040 cm^–1^ from the polymer backbone. These peaks are part of the polymer and should remain, but small shifts may occur due to environmental changes (from presence vs absence of Aβ1–42). The Amide I, C=O stretching of peptide bonds from proteins, at ∼1650 cm^–1^ is strong in the presence of Aβ1- 42, after desorption of the template molecule it was significantly reduced. The remaining absorption features, such as C=O stretching around 1700–1720 cm^–1^, C–H stretching bands at 2850–2950 cm^–1^, and C–N/C–O vibrations between 1000–1200 cm^–1^, correspond to the pyrrole-3-carboxylic acid–based polymer and PAMAM dendrimer framework, indicating the stability of the electrode modification. These spectral differences validate the formation of well-defined molecular imprints capable of selectively recognizing Aβ1–42. The sharp decrease of this peak after desorption indicates that desorption was successful.

**Figure 5:**
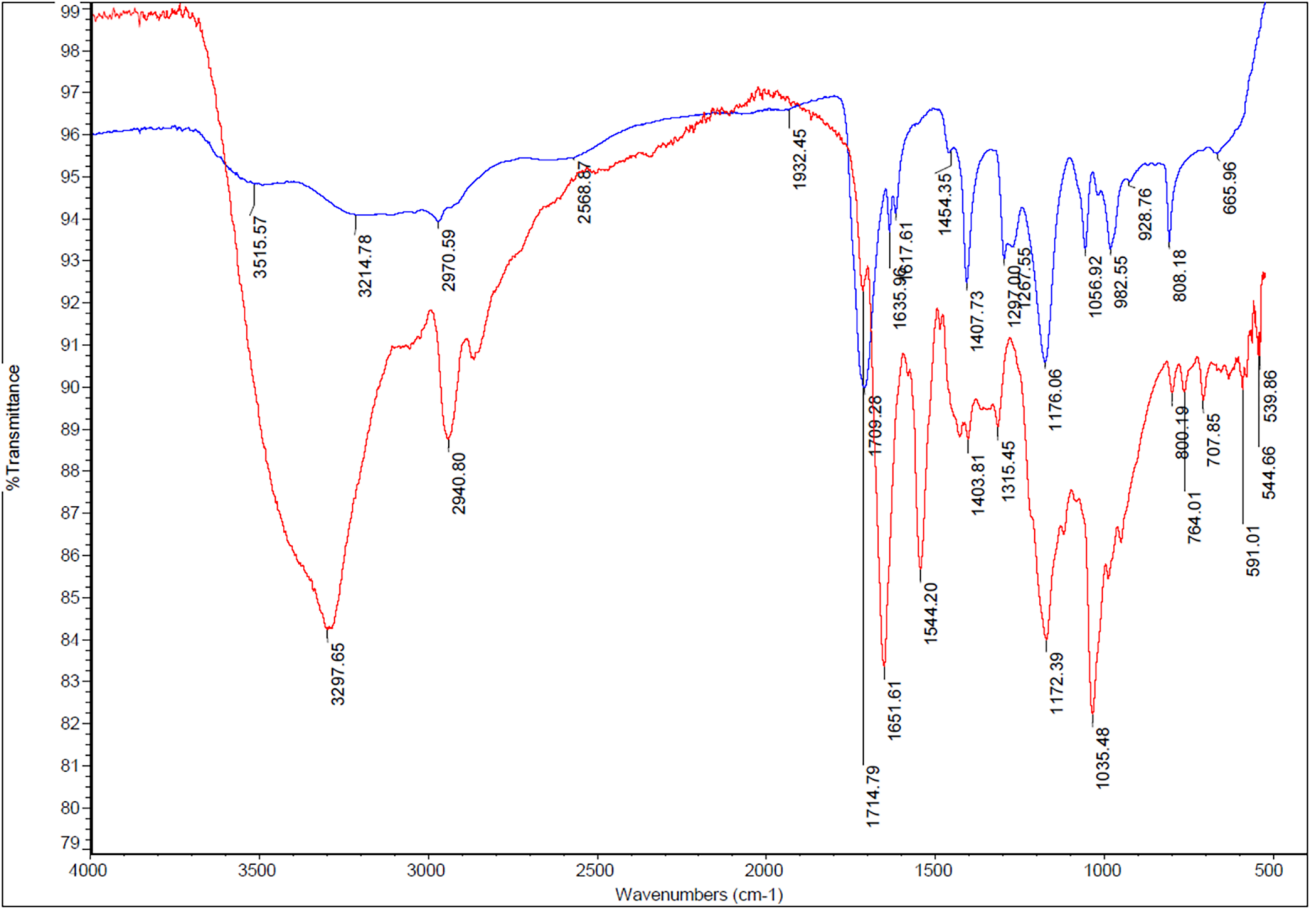
FT-IR spectra of Au-CYS-PAMAM- PPy3COOH- Aβ42 (Red) and Au-CYS-PAMAM- PPy3COOH (Blue) electrodes.

### 3.2 Performance of the Sensor

By measuring chronoimpedance, the optimal Aβ42 binding time of the molecularly imprinted electrode was determined. For 1000 s, the chronoimpedance was measured at 200 mV and 100 Hz. This involved first measuring the electrode’s chronoimpedance for 1000 s in a pH 7.4 50 mM phosphate buffer (green) and commercial serum (blue), followed by the addition of 50 μL of 66.7 ng/mL Aβ42 to the measurement cell and another 1000 s of chronoimpedance (red) (Figure 6A). 200 s was found to be the ideal binding time for the electrode made using the chronoimpedance test. The adsorption process was also performed in order to measure the fully cleaning of the electrode surface (Figure 6B) shown as light blue.

**Figure 6:**
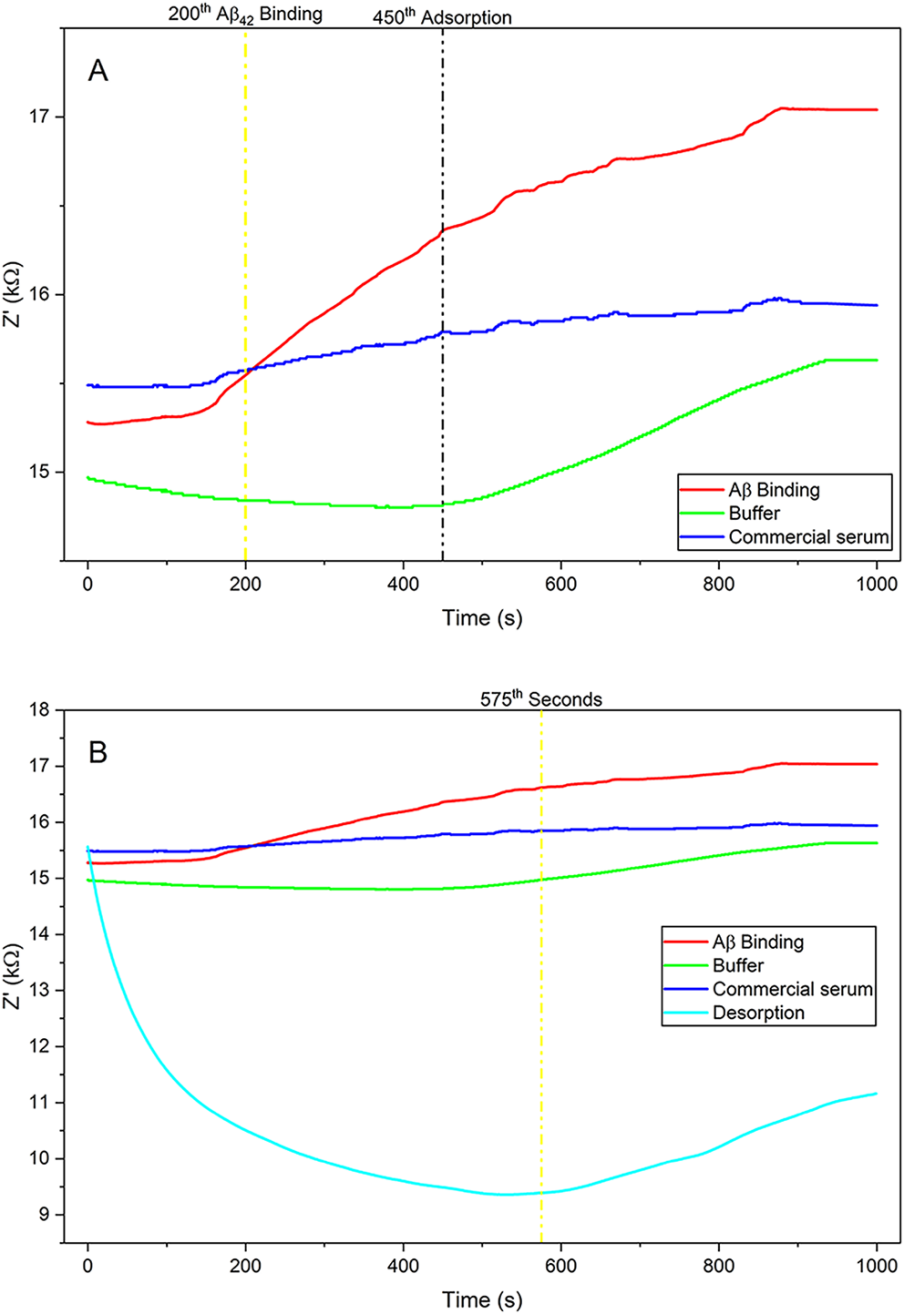
(A) Real-time impedance (*Z*′) monitoring of Aβ_1–42_ binding and subsequent adsorption processes on the modified electrode surface. The yellow dashed line marks the 200th second, corresponding to the initiation of Aβ_1–42_ binding, while the black dashed line at the 450th second represents the onset of nonspecific adsorption. (B) Real-time impedance (*Z*′) measurements illustrating Aβ_1–42_ binding, buffer response, commercial serum interaction, and the desorption process. The yellow dashed line marks the 575th second, corresponding to the end of the desorption step.

Figure 7 showed the calibration graph with the EIS measurements. Tests for LOD, LOQ, and repeatability were also conducted and are displayed in Table 2. A linear detection range of 0.5 to 20 ng/mL Aβ42 was found.

**Figure 7:**
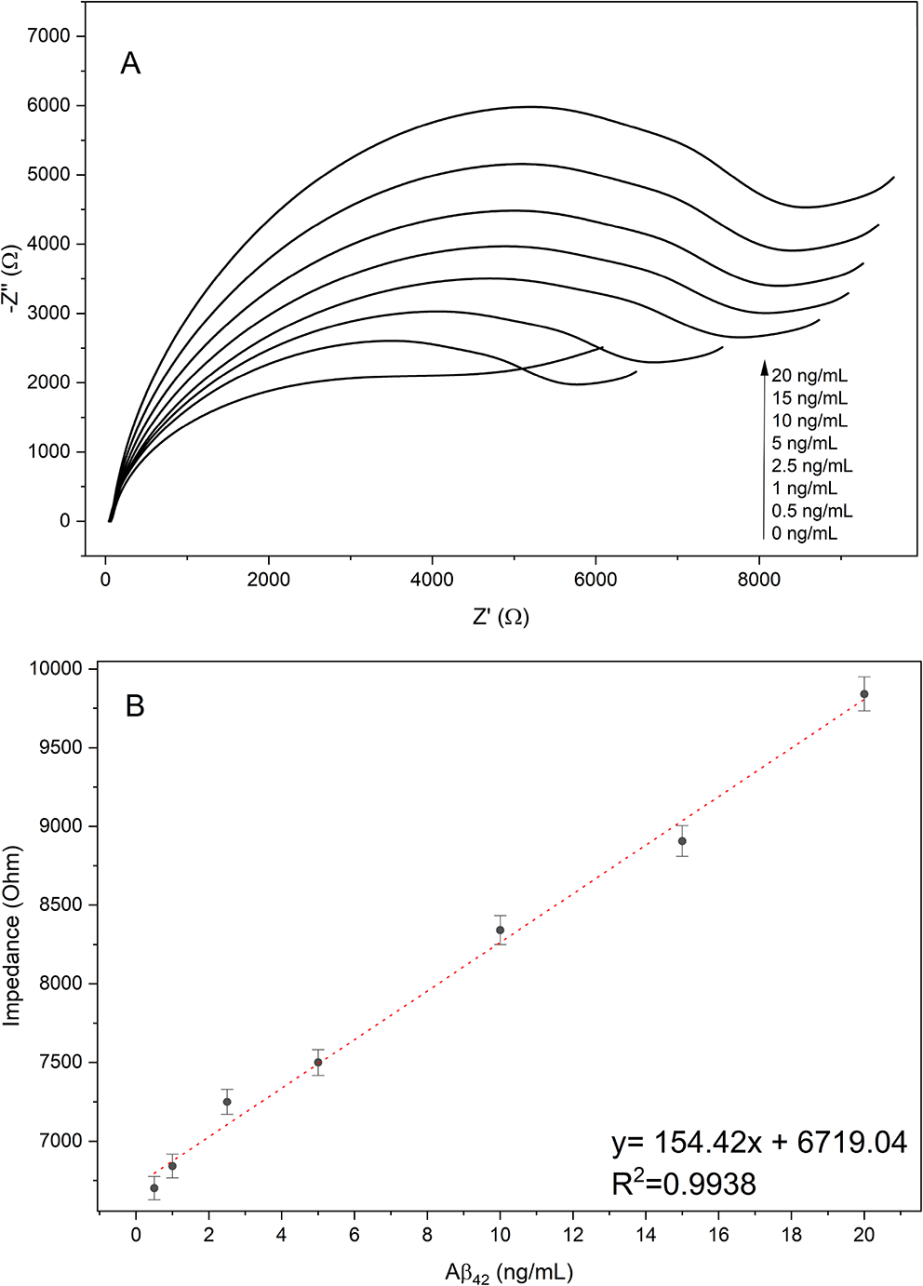
(A) Nyquist plots obtained for Aβ_1–42_ at increasing concentrations (0–20 ng/mL). The progressive enlargement of the semicircle diameter reflects the concentration-dependent increase in charge-transfer resistance upon peptide binding to the modified electrode surface. (B) Calibration curve generated from impedance values extracted at the characteristic frequency. A linear relationship is observed between impedance and Aβ_1–42_ concentration, described by the regression equation *y* = 154.42*x* + 6719.04 with *R* ^2^ = 0.9938, demonstrating excellent analytical linearity within the tested range.

**Table 2:** Performance Characteristics of the Aβ42 Sensor

linearity	0.5–20 ng/mL
*R* ^2^	0.9938
reproducibility (*n* = 4)	0.9938 ± 0.0062
repeatibility and recovery (*n* = 3) 5 ng/mL 20 ng/mL	5.54 (111%) 18.4 (92%)
LOD	0.14 ng/mL
LOQ	0.42 ng/mL

To verify the operability of the MIP electrode, the nonimprinted (NIP) electrode was carried out without adding the template molecule Aβ42 to the polymerization solution. The amount of each monomers were utilized similarly to the produced electrode that was imprinted. The NIP electrode was tested using the CI method and compared with the MIP with non response. Comparing the chronoimpedance data of the MIP and NIP sensors revealed that the NIP sensor was not responsive to Aβ42 within the imprinted sensor’s response range (Figure S3). The Aβ42 molecule deposited on the surface is responsible for the rise that occurs after ∼600 s. To evaluate the selectivity and potential interference effects of the proposed sensor, Tau protein—another clinically relevant biomarker associated with Alzheimer’s disease—was selected as an interfering species. The electrochemical impedance responses indicated that the presence of Tau did not produce a significant change compared to the target Aβ 1–42 signal. This result confirms that the developed MIP-based sensor possesses high selectivity toward Aβ 1–42. The observed selectivity is attributed to the specific recognition cavities formed during the molecular imprinting process, which are complementary in shape, size, and functional groups to the target molecule, thereby minimizing nonspecific interactions. Chronoimpedance measurement was employed for this. As previously mentioned, two electrodes were first made. These electrodes were measured independently for 1000 s in 3 mL of pH 7.4 phosphate buffer (baseline). The experiment was then repeated for 1000 s after 10 μL of stock protein solutions were added to the measurement medium with no response (Figure S4).

To assess the sensor’s storage stability, six electrodes were prepared. Three of the electrodes were maintained at ambient temperature and three at +4 degrees beyond the initial measurement day. The storage stability of the fabricated sensor was evaluated over 30 days at both 4 °C and room temperature. The sensor retained 92 and 85% of its initial response after 15 and 30 days, respectively, when stored at 4 °C, indicating good long-term stability under refrigerated conditions. In contrast, a significant decrease in response was observed at room temperature, with signal retention dropping to 72 and 56% after 15 and 30 days, respectively. These results demonstrate that the proposed sensor should be stored at low temperatures to preserve its performance over time (Figure S5).

Commercial serum was used to test the sensor’s applicability. After adding Aβ42 to the serum using the conventional addition method, measurements were taken utilizing the devised sensor system (Table 3). And the results were compared with other sensor studies in Table 4.

**Table 3:** Real Sample Detection Performance of the Aβ42 Sensor

sample	110 ng/mL
measurement 1	118.2 ng/mL
measurement 2	120.0 ng/mL
measurement 3	113.3 ng/mL
mean	117.2 ng/mL
standard deviation	2.83 ng/mL

**Table 4:** Comparison of the Data from the Designed System with Reported Data in Literatüre

detection method	electrode	detection limit	references
EIS SWV	MCd/PANI/Au-SPE	0.25 ng/mL 0.20 ng/mL	^21^
Colorimetric	MIP/Glu/APTES/cellulose	0.71 ng/mL	^22^
EIS	antimAβ/proteinG/SAM/AuNPs-modified carbon DEP chip	0.57 nM	^23^
DPV	MIPs/PTH-MB-ERG/GCE	0.018 ng/mL	^24^
Potentiometry	MIP/SWCNTs	720 ng/mL	^25^
EIS	AuE–Cys–PAMAM–Aβ_1–42_–PPy_3_COOH	0.42 ng/mL	this work

## 4 Conclusion

Alzheimer’s disease is known to be biomarked by amyloid β. As a result, measuring these biomolecules is crucial. The aim in the biomedical sector is to measure, or more specifically, to make a measurement, at a low cost and without being influenced by physical elements. Therefore, while creating a new generation measurement, we carried out a study while keeping these crucial factors in mind. Gold electrode sensitivity data has been used to construct a novel type of amyloid β sensing device. PAMAM and more monomer than normal were used in this sensor to increase the SAM layer and surface area. The synergistic interactions between the functional components used in electrode production are responsible for the increased electrocatalytic activity (or electrochemical responsiveness for our purposes) toward Aβ (1–42). The PAMAM dendrimer is immobilized in a stable state by cross-linking it with glutaraldehyde after cysteamine provides an amine-terminated surface. Strong electrostatic interactions and hydrogen bonding with pyrrole-3-carboxylic acid and the Aβ (1–42) template are made possible by the PAMAM structure’s high density of amine groups. Pyrrole-3-carboxylic acid interacts with the amine groups of PAMAM and the functional groups of Aβ during electropolymerization, forming a conductive polymer network (1–42). These interactions facilitate the creation of distinct recognition sites and guarantee the template’s effective integration into the polymer matrix. These sites allow selective rebinding of Aβ (1–42) after template removal, which has a major impact on the charge transfer properties at the electrode interface. The high surface area and functional group density of PAMAM, along with the conductive polymer backbone, promote electron transmission and improve the electrochemical response. Therefore, the combined impacts of increased surface area, improved electron transport routes, and specific molecule identification are responsible for the improved electrocatalytic behavior.

The detection mechanism of Aβ 1–42 at the designed electrode is based on the molecularly imprinted polymer (MIP) recognition principle. During electropolymerization, Aβ 1–42 molecules act as templates and are embedded within the polymeric matrix formed by pyrrole-3-carboxylic acid in the presence of PAMAM dendrimers. After template removal, specific recognition cavities complementary in shape, size, and functional groups to Aβ 1–42 are created. Upon rebinding of Aβ 1–42, these cavities selectively capture the target molecule, leading to a change in the electrochemical response of the Fe­(CN)_6_^3–/4–^ redox probe. This change arises from the modulation of electron transfer at the electrode interface due to partial blocking of the electroactive surface. The presence of PAMAM enhances the sensitivity by increasing the density of functional groups and the effective surface area, facilitating stronger and more selective interactions with Aβ 1–42.

A significant portion of monomer was utilized to do the measurement at the nanogram level due to the enormous size of the amyloid β molecule. Consequently, this work produced high-selectivity next generation sensor systems for neurodegenerative disease diagnostics.

## Supplementary Material



## Data Availability

Data used is available throughout the manuscript text.

## Notes

Electrochemical circuit diagram of the electrode (Figure S1); XPS survey analysis of electrode preparation steps (Figure S2); comparison of Aβ42 NIP (red) and MIP (blue) sensor responses at the same concentration (Figure S3); selectivity of the sensor with Tau protein. Red) Response of the sensor to Aβ42 Blue) Response of the sensor for Tau protein (Figure S4); storage stability of the sensor (Figure S5); electrochemical surface area (ECSA) values calculated from CV measurements using the Randles–Sevcik equation (Table S1) (PDF)
