# Supplementary material for: Rapid Detection of Amyloid β1–42 via PAMAM G4 Supported Molecularly Imprinted Sensor
Source: ACS Omega. 2026 Apr 18;11(17):25960–8. doi: 10.1021/acsomega.6c01809 (PMC13150757; doi:10.1021/acsomega.6c01809)
Supplement: Supplementary file 1 [file ao6c01809_si_001.pdf]

Rapid Detection of Amyloid β1-42 via PAMAM G4 Supported Molecularly Imprinted Sensor

Hilmiye Deniz ERTUĞRUL UYGUN*[1], Münire Nalan DEMİR[2]

[1]Dokuz Eylül University, Center for Fabrication and Application of Electronic Materials, Izmir 35390, Turkey

[2] Dokuz Eylül University, Faculty of Science, Department of Chemistry, Izmir 35390, Turkey

*corresponding author: deniz.uygun@deu.edu.tr

Contents of Supplementary Information

Figure S1. Electrochemical circuit diagram of the electrode

Figure S2. XPS survey analysis of electrode preparation steps

Figure S3. Comparison of Aβ42 NIP (red) and MIP (blue) sensor responses at the same concentration

Figure S4. Selectivity of the sensor with Tau protein. Red) Response of the sensor to Aβ42 Blue) Response of the sensor for Tau protein.

Figure S5. Storage stability of the sensor

Supplementary Table 1. Electrochemical surface area (ECSA) values calculated from CV measurements using the Randles–Sevcik equation

This supplementary file contains additional experimental data, characterization results, and supporting figures related to the main manuscript.

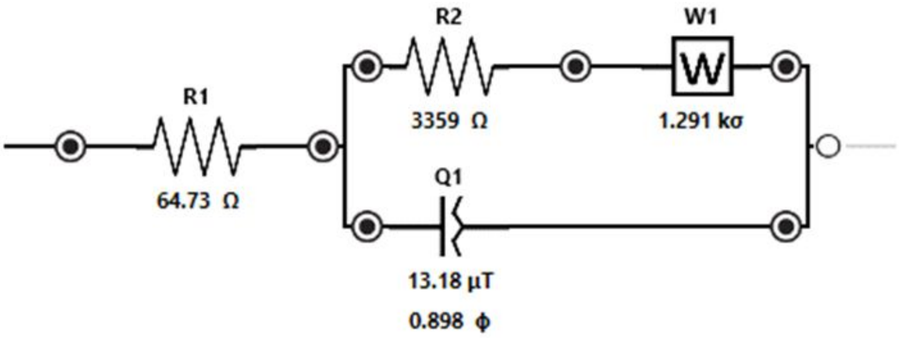

Figure S1. Electrochemical circuit diagram of the electrode

The circuit model is presented in Figure S1, where the data from EIS are represented in a circuit diagram according to the EIS curves. The elements of this circuit model: R1: resistance of the solution, R2: electron transfer resistance of the electrode surface, W1: Warburg impedance, Q1: Constant Phase Element

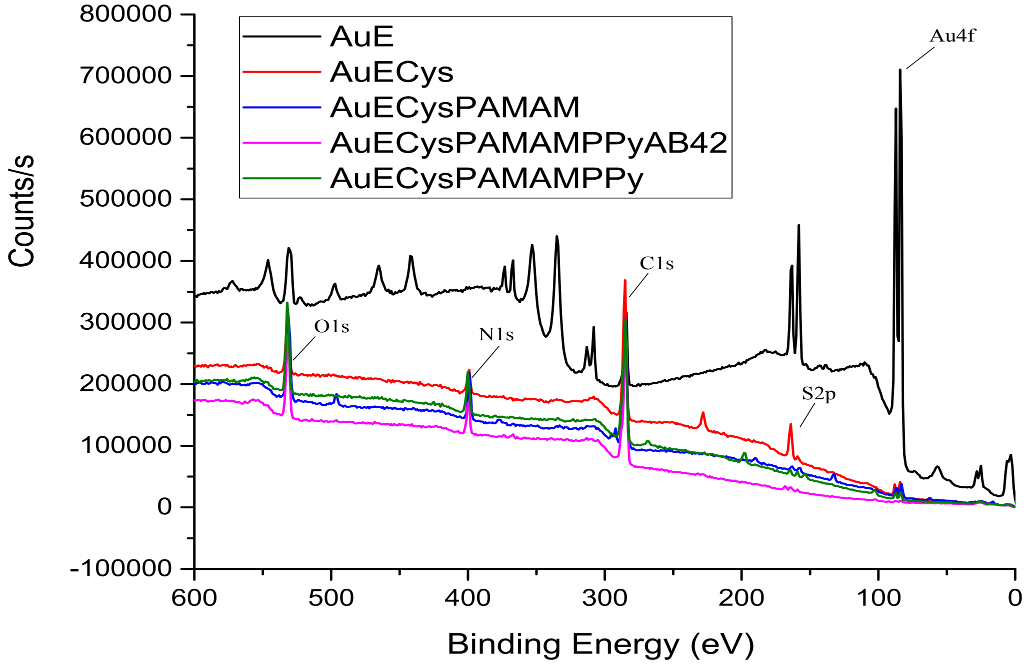

Figure S2. XPS survey analysis of electrode preparation steps

The XPS survey results for each modification step are given separately below.

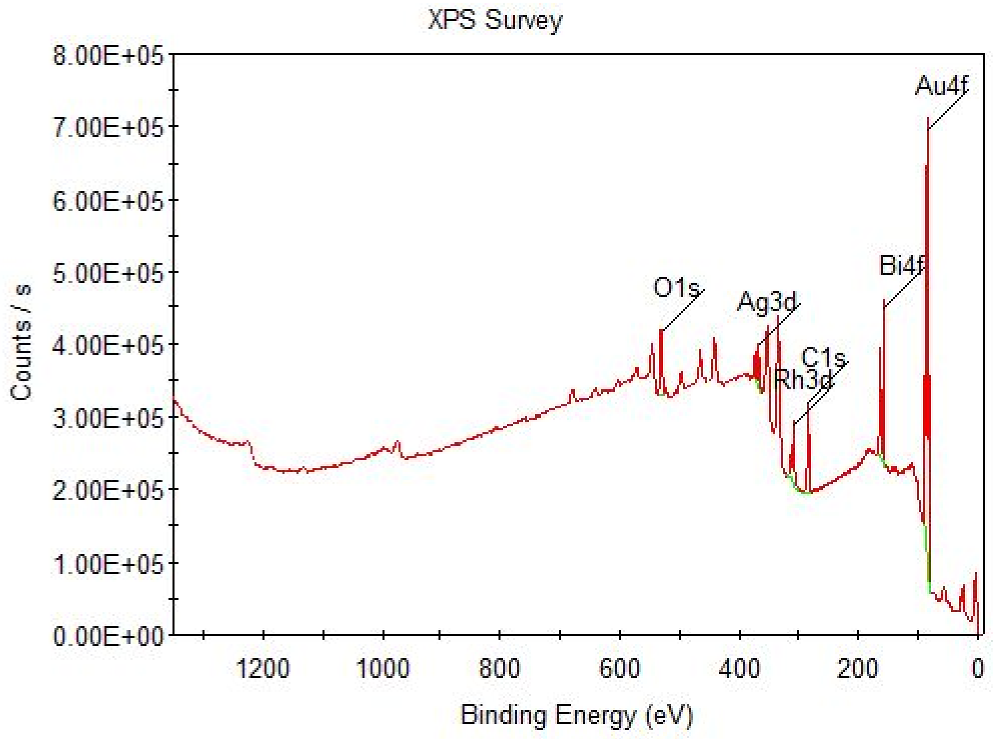

a. AuE

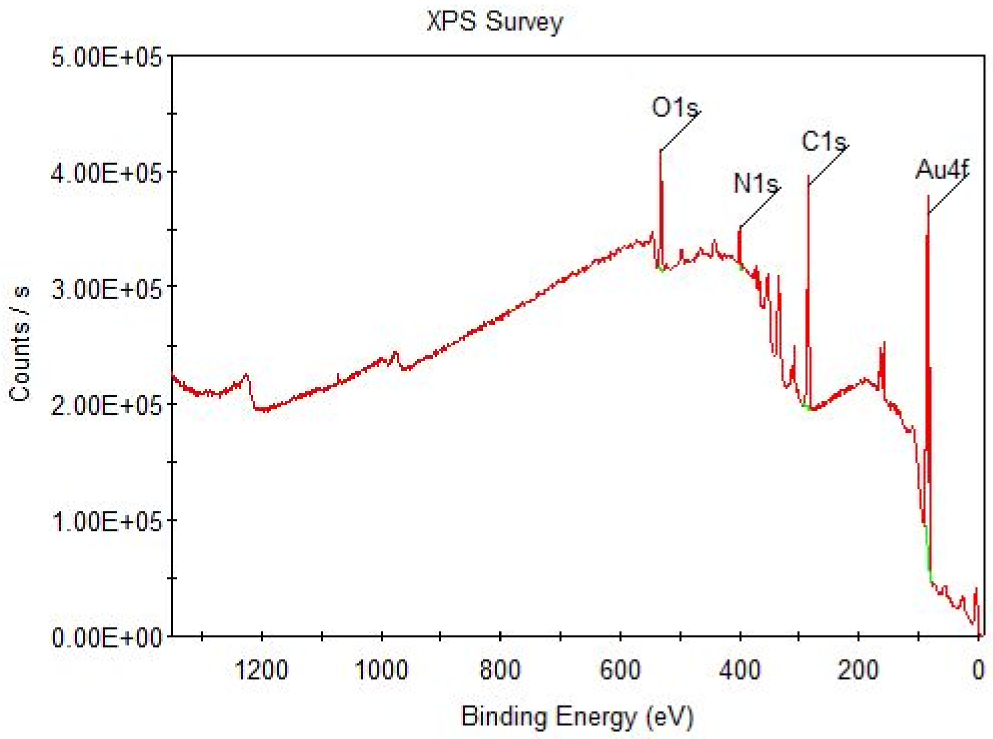

b. AuE-CYS

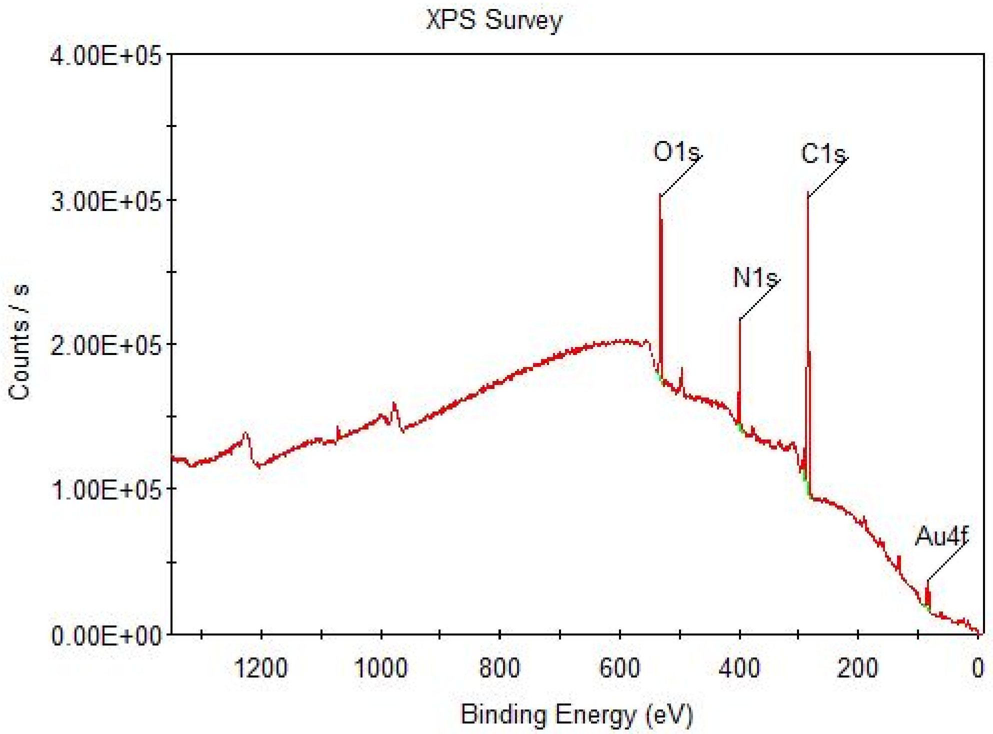

c. AuE-CYS-PAMAM

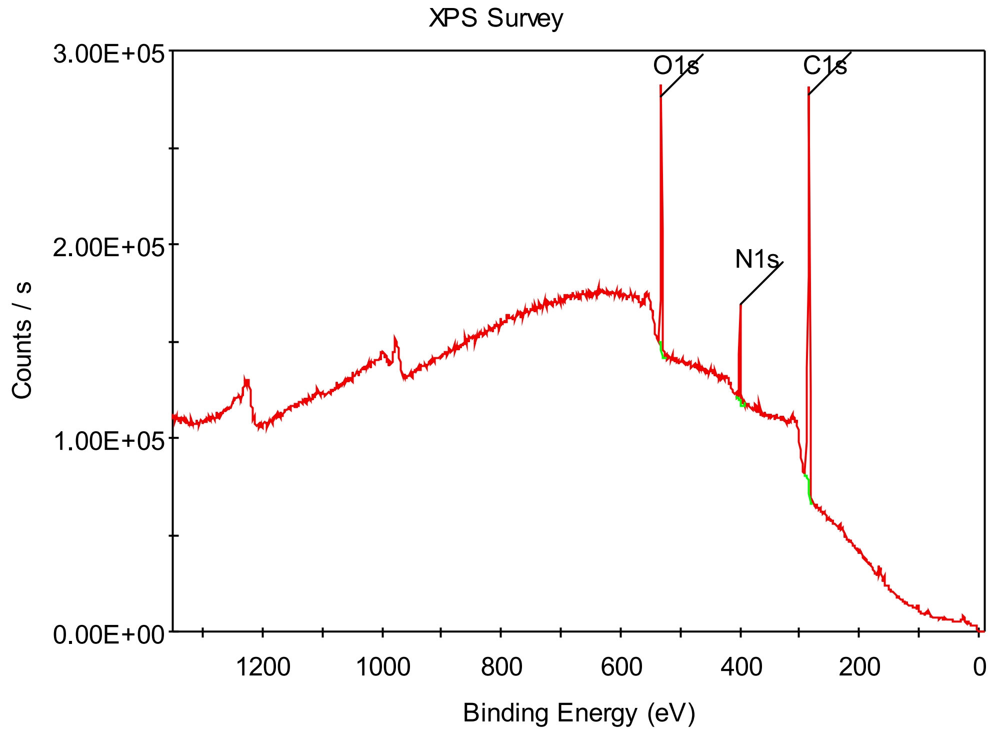

d. AuE-CYS-PAMAM-PPy-Aβ42

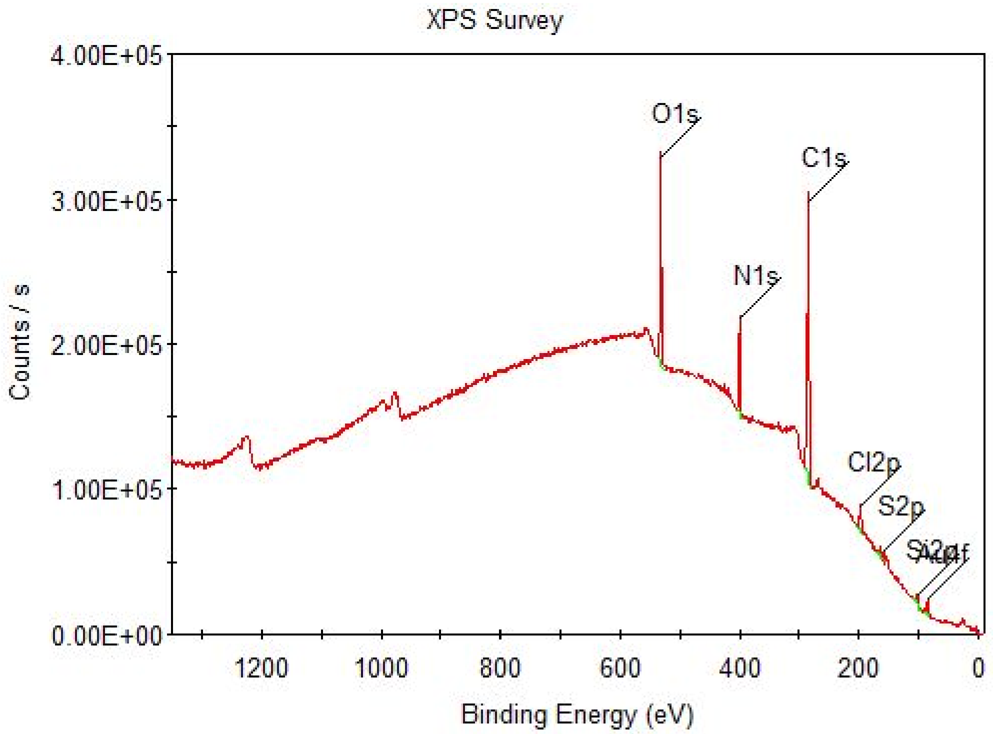

e. AuE-CYS-PAMAM-PPy (After desorption of Aβ42)

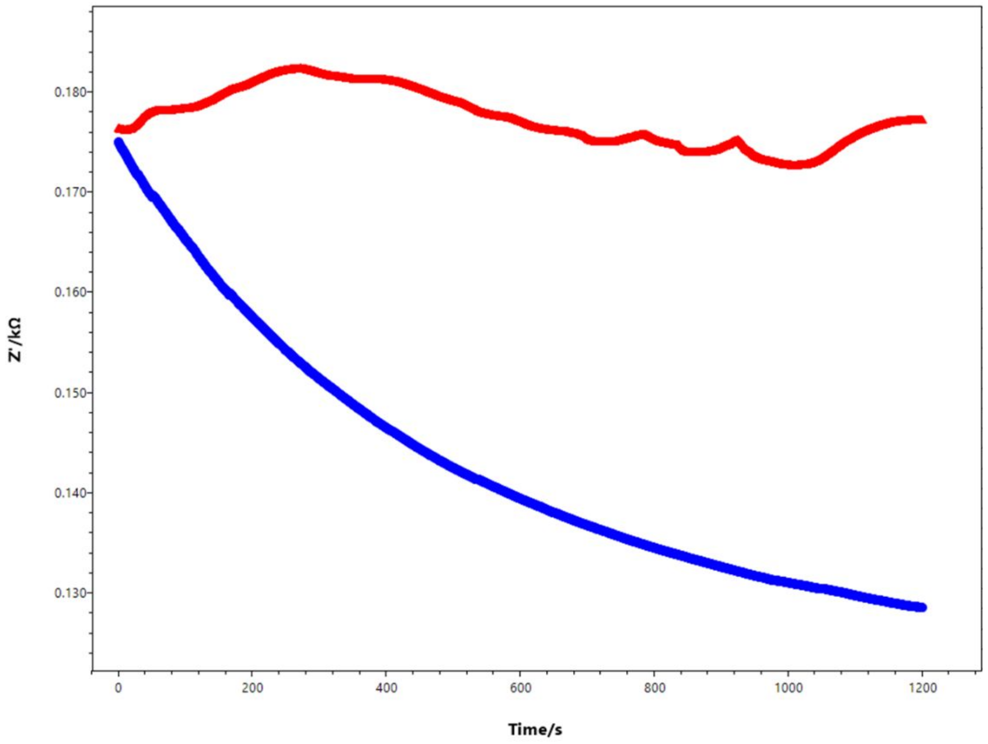

Figure S3. Comparison of Aβ42 NIP (red) and MIP (blue) sensor responses at the same concentration

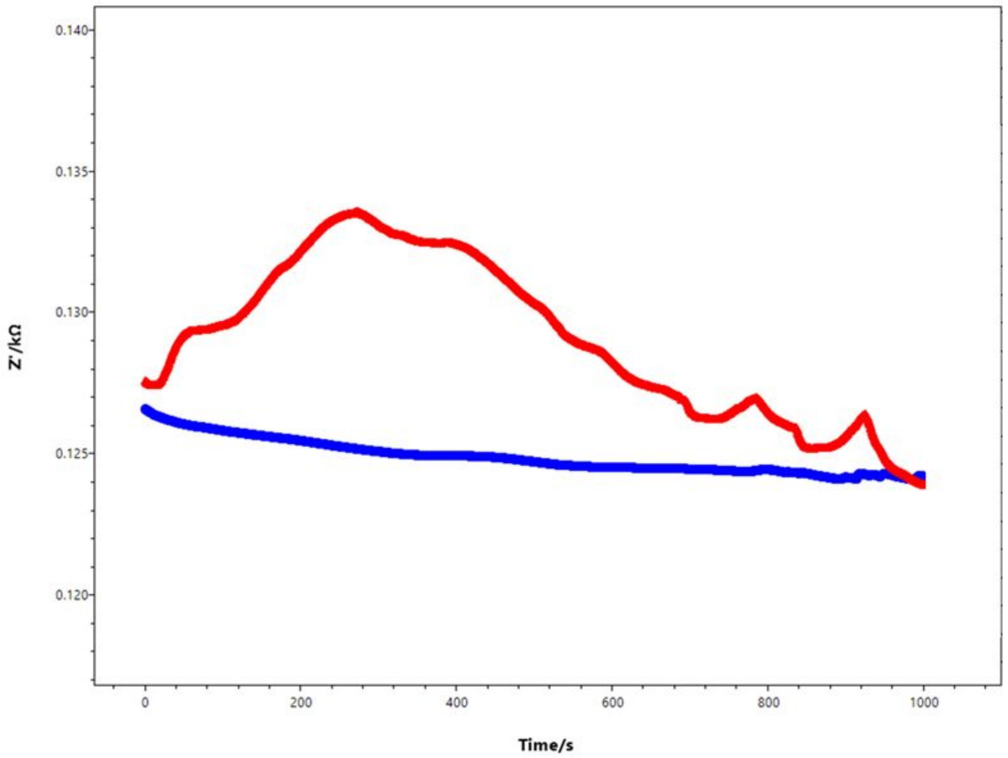

Figure S4. Selectivity of the sensor with Tau protein. Red) Response of the sensor to Aβ42 Blue) Response of the sensor for Tau protein.

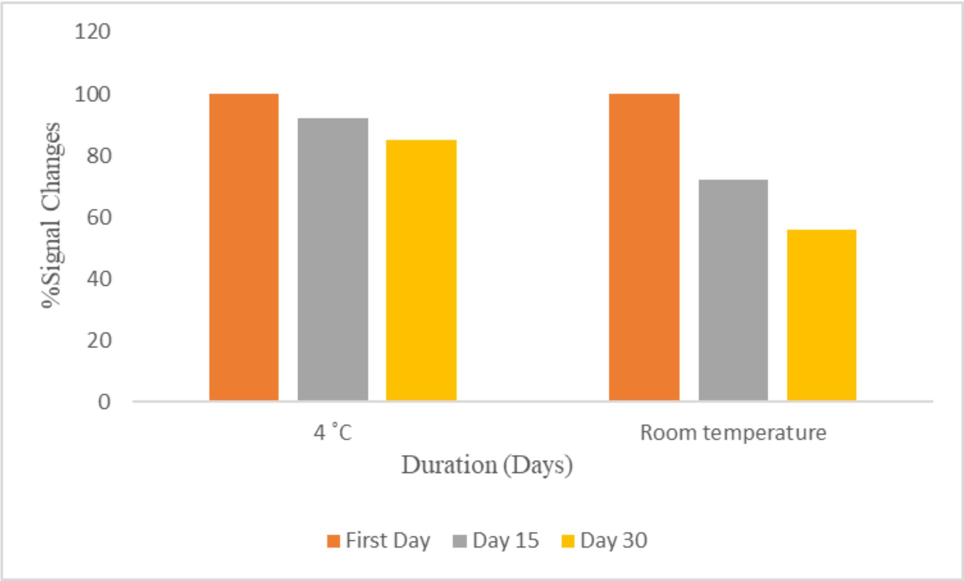

Figure S5. Storage stability of the sensor

Supplementary Table 1. Electrochemical surface area (ECSA) values calculated from CV measurements using the Randles–Sevcik equation

Electrode	Peak Current, Ip (μA)	ECSA (cm^2)
AuE	140	0.0120
AuE-Cys	150	0.0128
AuE-Cys-PAMAM	185	0.0158
AuE-Imp	25	0.0021
AuE-Desorption	50	0.0043

$$I_p = 2.69x10^5 AD^{1/2}n^{3/2}r^{1/2}C$$

where I_p (A) is the peak current obtained from cyclic voltammetry, n is the number of electrons transferred in the redox process (for $Fe(CN)_6^{3-}/^{4-}$, $n = 1$), A (cm^2) is the electrochemical surface area of the electrode, D ($cm^2\ s^{-1}$) is the diffusion coefficient of the redox probe (7.6×10^{-6} cm^2 s^{-1} for $Fe(CN)_6^{3-}/^{4-}$), C (mol cm^{-3}) is the concentration of the redox species, and v (V s^{-1}) is the scan rate.
